# Sun-Induced Severe Symptomatic Hypercalcemia in a Patient With Sarcoidosis: A Case Report Highlighting the Importance of Patient Education and Early Recognition

**DOI:** 10.7759/cureus.92176

**Published:** 2025-09-12

**Authors:** Mortada Mohammed, Leena Abdelmoti, Khadija Mohamed, Lina Abdeldaim, Ammar Ahmed, Moneeb Mustafa

**Affiliations:** 1 Internal Medicine, Sentara Albemarle Medical Center, Elizabeth City, USA; 2 Internal Medicine, National Rehabilitation Hospital, Dublin, IRL; 3 Internal Medicine, University of Khartoum, Khartoum, SDN; 4 Internal Medicine, Mayo Clinic, Rochester, USA; 5 Medicine, University of Minnesota Medical School, Minneapolis, USA; 6 Internal Medicine, Edward Via College of Osteopathic Medicine, Monroe, USA; 7 Internal Medicine, Rapides Regional Medical Center, Alexandria, USA

**Keywords:** calcidiol, calcitriol-mediated hypercalcemia, granuloma, hypercalcemia., sarcoid granuloma, sarcoidosis

## Abstract

Sarcoidosis is a multisystem inflammatory disorder characterized by non-caseating granulomas that can disrupt calcium metabolism through extrarenal production of calcitriol. In affected individuals, sun exposure may precipitate severe symptomatic hypercalcemia. We present the case of a 46-year-old male patient who initially presented with acute foot pain and was incidentally found to have extensive lymphadenopathy. Biopsy confirmed sarcoidosis, but the patient was lost to follow-up. One year later, after a two-week sun holiday, he developed severe hypercalcemia and acute kidney injury, presenting with fatigue, hypersomnia, and gastrointestinal symptoms. Standard treatments for hypercalcemia were ineffective, but rapid clinical improvement occurred with the initiation of corticosteroid therapy. This case highlights the importance of educating patients with sarcoidosis about the risks of sun exposure and underscores the necessity of disease-specific treatment, as corticosteroids are crucial for managing sarcoidosis-induced hypercalcemia.

## Introduction

Sarcoidosis is a multisystem inflammatory disorder of unknown etiology, characterized by the formation of non-caseating granulomas in affected organs. The condition affects approximately 5-40 per 100,000 individuals in European populations [1,2], with a reported prevalence of 28.13 per 100,000 in Ireland [3]. While pulmonary manifestations predominate [4,5], sarcoidosis can affect virtually any organ system [6], leading to diverse clinical presentations that often challenge diagnostic acumen.

One of the most clinically significant extrapulmonary manifestations is dysregulated calcium metabolism, occurring in approximately 5-10% of patients with sarcoidosis [7]. This complication arises from the aberrant extrarenal production of 1,25-dihydroxyvitamin D₃ (calcitriol) by activated macrophages within granulomas. These macrophages express 1α-hydroxylase, the enzyme responsible for converting 25-hydroxyvitamin D₃ (calcidiol) to the active hormone calcitriol, independent of normal parathyroid hormone (PTH) regulation and negative feedback mechanisms [8,9].

The clinical significance of this pathophysiology becomes particularly apparent during periods of increased vitamin D substrate availability, such as following sun exposure or vitamin D supplementation [10]. The resulting hypercalcemia can manifest with nonspecific symptoms that may be mistaken for other conditions, particularly heat-related illness in patients returning from sunny vacations [11]. This case report illustrates the potentially life-threatening consequences of unrecognized sarcoidosis-associated hypercalcemia and emphasizes the critical importance of patient education and clinical vigilance.

## Case presentation

A 46-year-old Caucasian male patient initially presented to the emergency department with an acute onset of severe left foot pain along the medial longitudinal arch that awakened him from sleep. The pain was severe (7/10 on a numeric rating scale) and prevented weight-bearing. Physical examination revealed erythema and marked edema of the affected area. He had no significant past medical history except for a chronic, asymptomatic, hypopigmented macular lesion on his right medial and posterior thigh.

**Figure 1:**
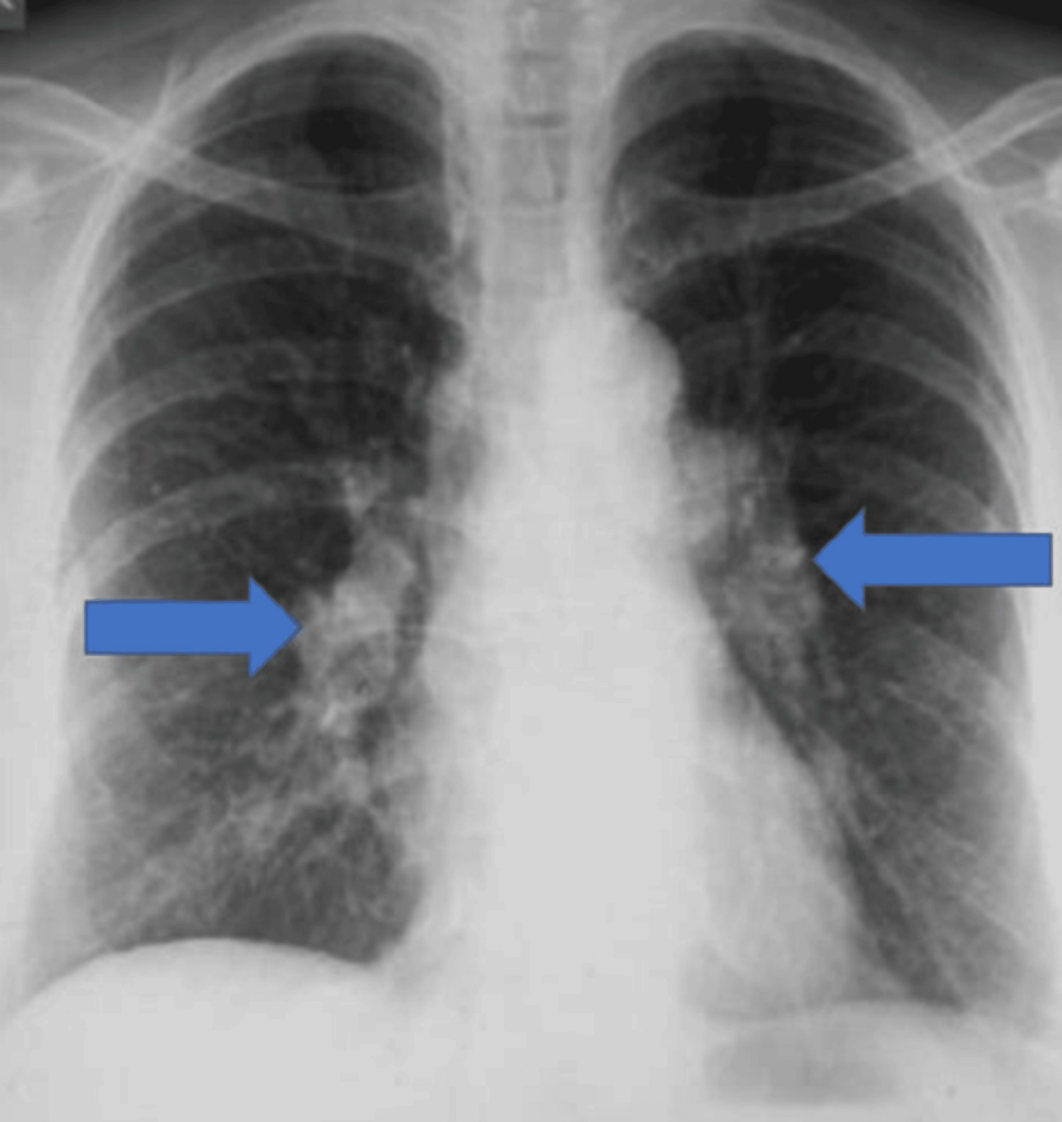
Chest X-Ray at the time of the initial presentation Posteroanterior (PA) chest radiograph demonstrating bilateral hilar lymphadenopathy (arrows) with mediastinal widening

**Figure 2:**
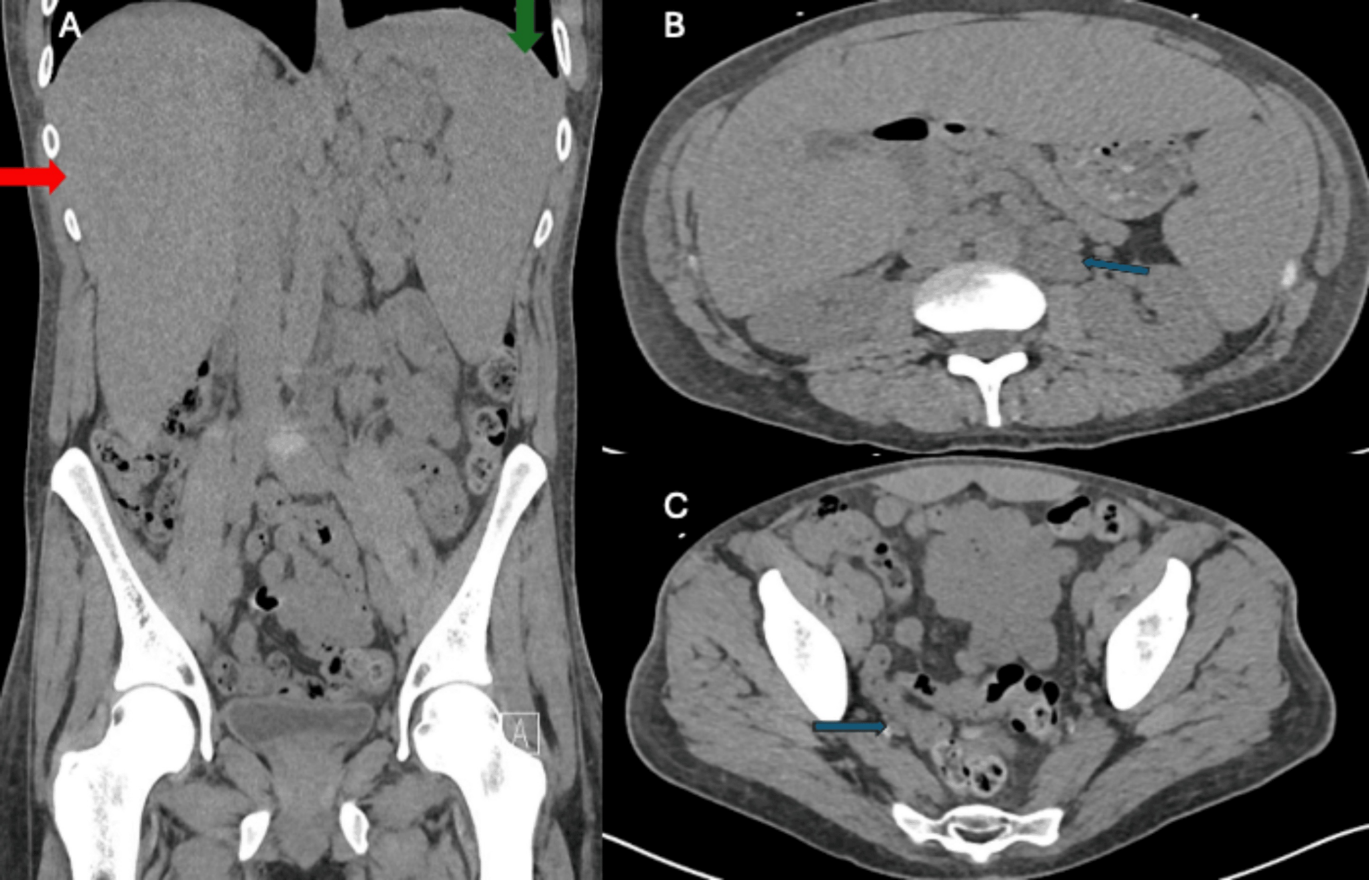
Non-contrast CT imaging demonstrating hepatosplenomegaly and abdominopelvic lymphadenopathy (A) Coronal view showing hepatomegaly (red arrow) and splenomegaly (green arrow). (B) Axial view highlighting an enlarged left para-aortic infrarenal lymph node (blue arrow), measuring approximately 19 × 28 mm. (C) Axial pelvic section showing an enlarged right external iliac chain lymph node (blue arrow), measuring approximately 15 × 24 mm.

Plain radiographs of the foot showed no acute abnormalities. However, a routine chest X-ray revealed bilateral hilar lymphadenopathy and mediastinal widening (Figure 1). CT imaging of the chest, abdomen, and pelvis revealed extensive lymphadenopathy extending from the supraclavicular to the inguinal regions, as well as hepatosplenomegaly (Figure 2). An inguinal lymph node biopsy revealed non-caseating granulomas consistent with sarcoidosis. Unfortunately, the patient was lost to follow-up and received no further treatment.

Approximately one year later, during the summer months, the patient presented to the emergency department again with a two-week history of progressive symptoms that began following his return from a two-week sun holiday in a Mediterranean destination. His presenting complaints included severe fatigue, hypersomnia (sleeping up to 20 hours per day), light-headedness, marked reduction in concentration, complete loss of appetite, nausea, vomiting, severe constipation, and confusion.

Treatment and clinical course

Initial management followed standard hypercalcemia protocols with aggressive IV fluid resuscitation (normal saline 4-6 L/day) and furosemide 40 mg IV twice daily. Despite 48 hours of intensive conventional treatment, calcium levels remained persistently elevated at 3.35 mmol/L with no clinical improvement.

Recognizing the underlying sarcoidosis and the mechanism of hypercalcemia, oral prednisolone was initiated at 40 mg daily. Within 24 hours of initiating corticosteroids, the patient's serum calcium level decreased to 2.95 mmol/L, and they reported improved mental clarity and reduced fatigue. By day 3, the calcium level decreased to 2.75 mmol/L, accompanied by marked clinical improvement. By day 7, the calcium level had normalized to 2.30 mmol/L, allowing for discharge home.

The patient was discharged on prednisolone 40 mg daily with close outpatient monitoring. One week post discharge, symptoms had completely resolved, serum calcium was normal (2.25 mmol/L), and renal function was improving (eGFR 55 mL/min/1.73 m²) (Table 1). A gradual steroid taper was implemented over a three-month period, accompanied by extensive patient education on the risks of sun exposure and the importance of sun protection measures.

**Table 1:** Laboratory parameters over the course of presentation and management eGFR, estimated glomerular filtration rate; PTH, parathyroid hormone; 25(OH)D₃, 25-hydroxyvitamin D₃ (calcidiol); 1,25(OH)₂D₃, 1,25-dihydroxyvitamin D₃ (calcitirol).

Parameter	Reference Range	On presentation	Day 3 Treatment	Follow-up (1 week)
Corrected Calcium (mmol/L)	2.12–2.62	3.48	2.75	2.25
eGFR (mL/min/1.73m²)	>90	40	45	55
Creatinine (μmol/L)	60–110	180	155	125
Phosphate (mmol/L)	0.87–1.45	0.8	0.9	1.1
PTH (pmol/L)	1.6–6.9	<0.5	<0.5	1.2
25(OH)D₃ (nmol/L)	75–200	95	Not measured	85
1,25(OH)₂D₃ (pmol/L)	43–144	285	Not measured	155
Albumin (g/L)	35–50	38	39	40
Hemoglobin (g/L)	130–180	112	118	125

## Discussion

This case exemplifies the complex calcium metabolism disturbances that can occur in sarcoidosis, highlighting several critical clinical lessons. The pathophysiology involves aberrant extrarenal production of calcitriol by activated macrophages within granulomatous tissue [9]. These macrophages express high levels of 1α-hydroxylase (CYP27B1), a cytochrome P450 enzyme found in renal proximal tubular cells, which converts the relatively inactive calcidiol to the potent hormone calcitriol [12,13].

In normal physiology, calcitriol production is tightly regulated by PTH, serum phosphate levels, and a negative feedback loop involving serum calcium. However, in sarcoidosis, the granulomatous production of calcitriol operates independently of these regulatory mechanisms [14]. Consequently, increased substrate availability from sun exposure can lead to uncontrolled calcitriol production and subsequent hypercalcemia.

The temporal relationship between sun exposure and hypercalcemic crisis in this case is particularly noteworthy. Several previous reports have documented similar presentations in sarcoidosis patients who have traveled to sunny destinations [15,16]. The Mediterranean holiday in this case likely provided sufficient UV-B radiation to increase endogenous vitamin D₃ synthesis significantly [17], leading to elevated calcidiol levels and subsequent uncontrolled conversion to calcitriol by granulomatous tissue. This mechanism explains why conventional hypercalcemia treatments (fluid resuscitation and loop diuretics) were ineffective, as the ongoing production of calcitriol continued to drive calcium absorption.

The clinical presentation of hypercalcemia can be notably nonspecific, often described by the mnemonic "stones, bones, groans, and psychiatric overtones" [18]. In the context of recent sun exposure, symptoms such as fatigue, confusion, and gastrointestinal upset can be easily attributed to heat exhaustion, potentially delaying an appropriate diagnosis and treatment [19]. Key clinical clues that should raise suspicion include a history of sarcoidosis, recent sun exposure, hypercalcemia with suppressed PTH levels, elevated calcitriol levels, and failure to respond to conventional treatment.

The cornerstone of treatment for sarcoidosis-associated hypercalcemia is corticosteroid therapy, which directly addresses the underlying pathophysiology by suppressing granulomatous inflammation and reducing macrophage 1α-hydroxylase activity [20]. The rapid response observed in this case following prednisolone initiation is characteristic and supports the diagnosis. Conventional treatments for hypercalcemia may provide some benefit, but are often insufficient as monotherapy because they do not address the ongoing calcitriol production.

This case highlights the crucial role of patient education in managing sarcoidosis. Key educational points include sun exposure precautions (using high-sun protection factor (SPF) sunscreen, protective clothing, and limiting sun exposure during peak UV hours), recognizing symptoms of hypercalcemia, caution regarding vitamin D supplementation, and the importance of regular monitoring. The initial loss to follow-up in this case nearly resulted in a life-threatening complication that could have been prevented with appropriate patient education and monitoring.

## Conclusions

This case demonstrates that sun exposure can precipitate life-threatening hypercalcemia in sarcoidosis patients through aberrant granulomatous calcitriol production, where conventional treatments fail and corticosteroids are essential. The nonspecific presentation can easily be mistaken for heat-related illness, highlighting the diagnostic challenge clinicians face.

Patient education on sun protection, adequate follow-up care, and increased awareness among healthcare providers are critical to preventing this potentially fatal but treatable complication. This case underscores how loss to follow-up can lead to preventable medical emergencies in sarcoidosis patients.
